# Functional Characterization of a Signal Peptide Peptidase in *Phaffia rhodozyma* Reveals a Potential Role in Protein Stress Response but Not in Activation of the SREBP Ortholog Sre1

**DOI:** 10.3390/ijms27062628

**Published:** 2026-03-13

**Authors:** Marcelo Baeza, Melissa Gómez, Gabriela Apariz, Salvador Barahona, Jennifer Alcaíno

**Affiliations:** 1 Departamento de Ciencias Ecológicas, Facultad de Ciencias, Universidad de Chile, Santiago 7800003, Chile; mbaeza@uchile.cl (M.B.); melissa.gomez@ug.uchile.cl (M.G.); gabriela.apariz@ug.uchile.cl (G.A.); 2 Facultad de Ciencias, Universidad de Chile, Santiago 7800003, Chile; salvador@uchile.cl

**Keywords:** signal peptide peptidase, SREBP, RNA-seq, carotenoids, protein stress

## Abstract

Sterol regulatory element-binding proteins (SREBPs) regulate lipid homeostasis and coordinate sterol metabolism and carotenogenesis in the astaxanthin-producing yeast *Phaffia rhodozyma*. While Sre1, the SREBP ortholog, and the site-2 protease Stp1 have been identified as essential components of this pathway in *P. rhodozyma*, additional factors involved in Sre1 processing or regulation remain unknown. In *Aspergillus* species, a signal peptide peptidase contributes to the activation of the SREBP ortholog, raising the possibility of a similar role in this yeast. In this work, we identified and characterized the *P. rhodozyma* signal peptide peptidase (SppA) homolog. Sequence analysis, domain prediction, and phylogenetic analyses supported its classification within the SPP family of intramembrane aspartyl proteases. To evaluate its functional role, Δ*sppA* mutants were constructed in genetic backgrounds with constitutive Sre1 activity, including the *cyp61*^−^ mutant and a strain expressing the active form of Sre1 (Sre1N). Deletion of *SPPA* did not alter sensitivity to clotrimazole or cobalt chloride, nor affect pigmentation, indicating that SppA is not required for Sre1 activation in *P. rhodozyma*. Transcriptomic analyses further showed that expression of *SRE1* and of its known target genes remained unchanged upon *SPPA* deletion. Interestingly, the loss of SppA in the Sre1N background caused marked downregulation of genes associated with protein refolding and unfolded protein binding. In agreement with these transcriptional changes, the Sre1NΔ*sppA* strain displayed increased sensitivity to dithiothreitol. These findings suggest that, although SppA is not involved in Sre1 activation in *P. rhodozyma*, it may play a role in protein stress-related processes. Future studies will be required to define the molecular mechanisms underlying this role and its integration with protein homeostasis networks.

## 1. Introduction

Microbial systems that naturally accumulate high-value metabolites offer valuable opportunities to investigate the regulatory mechanisms controlling specialized biosynthetic pathways. One such microorganism is *Phaffia rhodozyma* (*Xanthophyllomyces dendrorhous*), a basidiomycete carotenogenic yeast capable of producing the xanthophyll astaxanthin. Astaxanthin is a highly demanded carotenoid due to its applications in aquaculture, nutrition, and cosmetic industries [1,2,3]. In *P. rhodozyma*, carotenoids derive from the mevalonate (MVA) pathway, the same route that provides the universal isoprenoid precursors for sterol biosynthesis [4]. While the enzymatic steps of astaxanthin production are well established, much less is known about its regulation and how carotenoid synthesis is transcriptionally coordinated with sterol metabolism. A key insight into this connection came from the discovery that carotenoid biosynthesis is regulated by an SREBP (Sterol Regulatory Element-Binding Protein) ortholog [5,6].

SREBPs are membrane-bound transcription factors that coordinate lipid and sterol homeostasis across eukaryotes. They are synthesized as inactive precursors anchored to the endoplasmic reticulum (ER) membrane and require regulated proteolysis to release the N-terminal transcription factor domain that activates lipid biosynthetic genes [7,8,9]. In mammals, the SREBP precursor is retained in the endoplasmic reticulum through its interaction with SCAP (SREBP Cleavage-Activating Protein). This ER-resident intramembrane protein functions both as an escort for SREBP and sterol sensor [10,11]. SCAP function is controlled by INSIG (Insulin-Induced Gene) proteins, which bind SCAP under sterol-sufficient conditions, retaining the SCAP-SREBP complex in the ER. When sterol levels decline, SCAP undergoes a conformational change that disrupts its interaction with INSIG, allowing the complex to traffic to the Golgi apparatus [11,12,13]. Once in the Golgi, SREBPs are sequentially processed by two proteases: first by site-1 protease (S1P), a luminal subtilisin-like serine protease, and then by site-2 protease (S2P), a zinc-dependent intramembrane metalloprotease of the M50 family [14,15,16,17]. Following S2P processing in the mammalian pathway, the N-terminal bHLH-ZIP domain is released from the membrane which enters the nucleus and promotes transcription of genes required for sterol and fatty-acid biosynthesis [7,8,9].

In fungi, orthologs of SREBP and associated regulatory components (hereafter referred as “the SREBP pathway”) have been identified in several species. However, their organization and proteolytic mechanisms differ from the mammalian SCAP-INSIG-S1P/S2P system [6,7]. In the ascomycete fission yeast *Schizosaccharomyces pombe*, clear homologs of SREBP, SCAP, and INSIG (named Sre1, Scp1, and Ins1, respectively) have been described; however, only Sre1 and Scp1 participate in the pathway, as Ins1 is dispensable for Sre1 activation [18]. Notably, no S1P or S2P homologs participate in Sre1 activation in this yeast. Instead, Sre1 processing relies on a mechanism involving the Golgi Dsc E3 ligase complex, which initiates cleavage of the membrane-embedded precursor [19,20], followed by intramembrane proteolysis mediated by the rhomboid protease Rbd2 [21]. These findings illustrate that fungi can preserve SCAP-like sterol sensing while employing distinct proteolytic machinery for activation of the SREBP ortholog. A different configuration is observed in the basidiomycete yeast *Cryptococcus neoformans*, which possesses functional homologs of Sre1 and Scp1 [22] and, in contrast to *S. pombe*, requires a site-2 protease (named Stp1) for intramembrane cleavage and activation of Sre1 [23]. Filamentous fungi from the genus *Aspergillus* add yet more diversity. In *Aspergillus fumigatus*, the SREBP ortholog SrbA is processed by the Dsc E3 ligase complex [24] and a rhomboid protease [25,26], similar to *S. pombe*, but no SCAP homolog has been identified in this species [27]. Moreover, studies in *Aspergillus nidulans* revealed that the signal peptide peptidase SppA, an ER intramembrane aspartyl protease, performs an additional cleavage step after Dsc-mediated processing being essential for SrbA activation [28].

In *P. rhodozyma*, the SREBP pathway has only recently begun to be characterized. The SREBP homolog Sre1 [29] and the M50-family intramembrane protease Stp1 [30] were identified as essential components of the pathway. Loss of either gene results in hypersensitivity to azoles and cobalt chloride, and reduced sterol and carotenoid content, particularly in genetic backgrounds that constitutively activate Sre1, such as the *cyp61*^−^ ergosterol mutant [29,30]. In addition, the expression of the constitutively active form of Sre1 (Sre1N) leads to increased carotenoid accumulation [29]. These findings suggest that Sre1 links sterol homeostasis to carotenogenesis in this yeast [6]. However, only these two components have been functionally defined so far, and similar to *Aspergillus*, no SCAP homolog has been identified in the *P. rhodozyma* genome [6]. Given that the *P. rhodozyma* SREBP pathway shares some similarities with that from *Aspergillus*, it raises the possibility that Sre1 activation may also rely on another protease, in addition to Stp1, to complete its processing.

Signal peptide peptidases (SPPs), as SppA in *Aspergillus*, are intramembrane aspartyl proteases that use a conserved GxGD catalytic motif to cleave peptides embedded within lipid bilayers [31]. These enzymes were initially characterized for their ability to cleave signal peptide fragments that remain in the endoplasmic reticulum membrane following the initial processing step carried out by signal peptidase [32,33], thereby contributing to ER proteostasis and membrane protein quality control. SPPs are part of a broader GxGD protease family that also includes SPP-like proteins (SPPLs) and presenilins [34]. While sharing the conserved catalytic architecture, these proteins exhibit distinct functional properties. Presenilins process type I transmembrane proteins as part of the gamma-secretase complex [35,36,37], whereas SPPLs, like SPPs, function as monomers or homodimers and target type II transmembrane proteins. However, SPPLs differ from SPPs in their substrate specificity and subcellular localization, often functioning in the Golgi apparatus or at the plasma membrane to regulate diverse physiological signaling pathways [34,38].

In this work, we identified and characterized a signal peptide peptidase homolog in *P. rhodozyma* (named SppA) and evaluated its potential involvement in Sre1 activation. Using genetic, phenotypic, and transcriptomic analyses in backgrounds with constitutive Sre1 activity, our results indicate that its gene product is not required for Sre1 activation and therefore does not function as a core component of the SREBP pathway in this yeast. Nevertheless, the loss of SppA was associated with transcriptional changes affecting genes related to protein folding and unfolding, particularly in the Sre1N background, which may reflect the involvement of SppA in protein stress-related processes.

## 2. Results and Discussion

Because the SppA protease plays an important role in the activation of the SREBP ortholog in *Aspergillus* species, we first sought to identify and characterize the *P. rhodozyma* homolog of this intramembrane aspartyl protease. SppA belongs to the GxGD-type intramembrane aspartyl protease family, which comprises presenilins and the SPP/SPPL subfamilies, all of which share conserved catalytic motifs but differ in membrane orientation and biological roles.

### 2.1. Identification and Bioinformatic Characterization of the P. rhodozyma SppA Gene

To identify the gene encoding a signal peptide peptidase in *P. rhodozyma*, BLASTp searches were initially performed against a local database of signal peptide peptidases using translated coding sequences (CDS) previously predicted from *P. rhodozyma* [5,39]. A single candidate gene (ID g1388) was identified and named *SPPA,* following the nomenclature used for signal peptide peptidases in *Aspergillus* species. A reciprocal BLASTp search of the local SPP dataset against the *P. rhodozyma* predicted proteome identified only g1388 above the defined similarity thresholds, indicating the absence of additional detectable SPP-like paralogs in the genome. To further validate the gene identity, the deduced protein sequence was subjected to BLASTp searches against curated NCBI protein databases. The best BLASTp hit of the deduced protein against the RefSeq protein database corresponded to an aspartic endopeptidase from *Mrakia frigida*, with an e-value of 5 × 10^−164^, 100% query coverage, and 57% sequence identity. In the Swiss-Prot database, the best hit was a signal peptide peptidase from *Homo sapiens*, with an e-value of 4 × 10^−70^, 90% query coverage, and 37% sequence identity.

**Figure 1:**
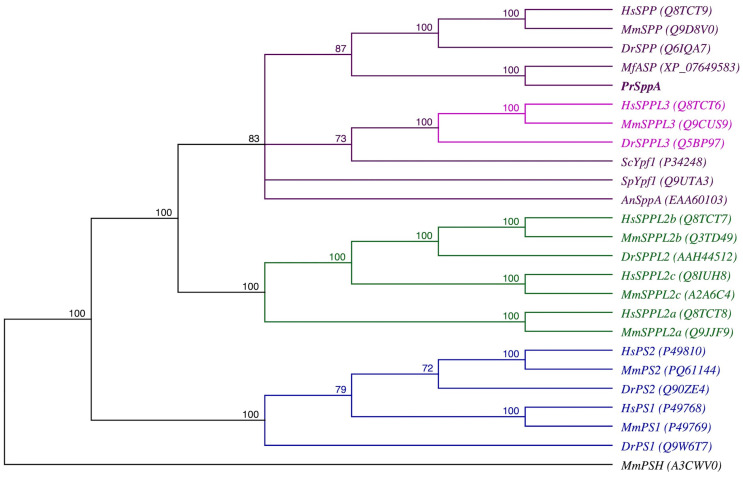
Phylogenetic placement of the *P. rhodozyma* signal peptide peptidase. Presilins (PS1 and PS2, blue); Signal peptide peptidases and fungal homologs (SPP, purple) and Signal peptide peptidases-like 3 proteins (SPPL3, pink); Signal peptide peptidases-like 2 proteins (SPPL2, green). Ypf1 (Yeast Presenilin-like Family); ASP (aspartyl protease); and PSH (presenilin homolog). Species: Hs, *Homo sapiens*; Mm, *Mus musculus*; Dr, *Danio rerio*; Mf, *Mrakia frigida*; An, *Aspergillus nidulans*; Pr, *Phaffia rhodozyma* (highlighted in bold); Sp, *Schizosaccharomyces pombe*; Sc, *Saccharomyces cerevisiae*. The archaeal PSH protein sequence from *Methanoculleus marisnigri* (MmPSH) was used as the outgroup (black). Numbers at the nodes indicate bootstrap support values calculated from 1000 replicates. Sequence accession codes are included in parentheses; the complete sequence alignment is provided in Figure S1.

To confirm the classification of the *P. rhodozyma* candidate sequence, a phylogenetic analysis of the GxGD intramembrane protease superfamily was performed. Two main groups can be distinguished in the resulting cladogram: one containing SPP and SPPL proteins and the other containing presenilins (Figure 1). Among the group containing SPP and SPPL proteins, SPPL3 appears to be closer to SPPs than SPPL2, as observed in other studies [40,41]. Interestingly, among the SPP and SPPL groups, the animal SPPs form a tight group due to their high similarity. In contrast, the included fungal sequences are more dispersed across separate branches; even the ascomycetes (*A. nidulans*, *S. cerevisiae*, *S. pombe*) proteins did not group, appearing in distinct branches within that group. Notably, the sequence from *P. rhodozyma* SppA as well as the aspartic endopeptidase from *M. frigida*, clustered with animal SPPs within the SPPs containing clade, clearly separated from presenilins and SPPL2s, although this topology is interpreted as a comparative placement rather than a comprehensive evolutionary inference. Consistently, BLASTp searches against the RefSeq database retrieved fungal homologs as the closest matches to the *P. rhodozyma* protein (best hit: an aspartic endopeptidase from *M. frigida*), indicating that the observed clustering does not necessarily reflect a preferential evolutionary relationship with metazoan SPPs.

**Figure 2:**
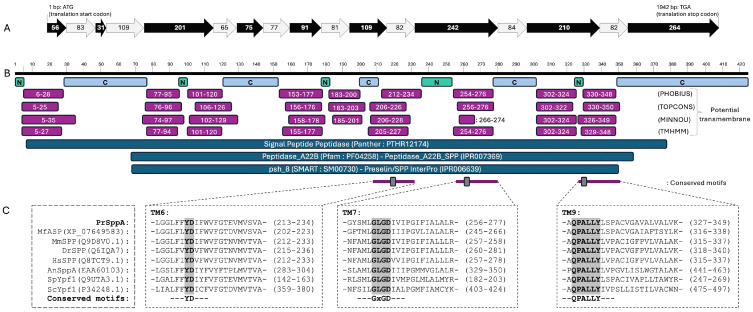
Bioinformatic analysis of the *P. rhodozyma SPPA* gene. (**A**) Gene structure of the *P. rhodozyma SPPA* locus. The gene consists of nine exons (black arrows) and eight introns (light gray arrows) and encodes a predicted protein of 425 amino acids. Numbers within exons and introns correspond to their nucleotide lengths, and the ATG start codon and TGA stop codon are indicated. (**B**) Predicted topology of the *P. rhodozyma* SppA protein. Transmembrane segments (purple, with residue positions indicated) were predicted using PHOBIUS [46], TOPCONS [47], MINNOU [48], and TMHMM [49]. Loop orientation predicted by PHOBIUS is shown in green (N: non-cytoplasmic) and light blue (C: cytoplasmic). Family classifications obtained from PANTHER [50], Pfam [51] and SMART [52] are shown below (blue). The conserved YD, GxGD, and QPALLY motifs located in TM6, TM7, and TM9, respectively, were manually identified and are highlighted in gray. (**C**) Multiple sequence alignment of TM6, TM7 and TM9 previously defined [53], using protein sequences from the SPP containing clade defined in Figure 1. The conserved YD, GxGD, and QPALLY motifs within their respective transmembrane helices are highlighted. For each species, the positions indicated correspond to the span of the transmembrane segments where these motifs reside. Ypf1 (Yeast Presenilin-like Family); ASP (aspartyl protease); and PSH (presenilin homolog). Species: Pr, *Phaffia rhodozyma;* Mf, *Mrakia frigida*; Mm, *Mus musculus*; Dr, *Danio rerio*; Hs, *Homo sapiens*; An, *Aspergillus nidulans*; Sp, *Schizosaccharomyces pombe*; Sc, *Saccharomyces cerevisiae* (sequence accession codes are shown in parentheses). The complete alignment is provided in Figure S1.

The *P. rhodozyma SPPA* gene spans 1941 bp (from start to stop codon) and contains nine exons, yielding an ORF of 1278 bp that encodes a predicted 425 amino acid protein (Figure 2 and Figure S2). InterPro analysis of the deduced protein sequence also strongly supports its identification as a signal peptide peptidase, with family-level hits to Presenilin/signal peptide peptidase (IPR006639) and Peptidase A22B, signal peptidase (IPR007369). Additional protein domain classifiers were consistent with this annotation: PANTHER identified the protein as a member of the Signal Peptide Peptidase family (PTHR12174; 87.3% coverage, e-value of 4 × 10^−93^), Pfam placed it in the Peptidase_A22B family (PF04258; 68.3% coverage, e-value of 9 × 10^−70^), and SMART assigned it to the psh_8 family (SM00730; 66.1% coverage, e-value of 3 × 10^−50^).

Functional annotation through PANTHER GO terms also supports the identity of SppA as a membrane-associated intramembrane aspartyl protease. Biological process terms include membrane protein proteolysis (GO:0033619) and signal peptide processing (GO:0006465). Molecular function terms include aspartic endopeptidase activity, intramembrane cleaving (GO:0042500), while cellular component annotations place the protein on both the luminal (GO:0098553) and cytoplasmic (GO:0098554) sides of the endoplasmic reticulum membrane. This predicted subcellular localization was further supported by analysis using the DeepLoc-2.1 web server, which assigned the protein to the endoplasmic reticulum with a probability of 0.79 and predicted transmembrane association with a probability of 0.95.

Topology prediction revealed that *P. rhodozyma* SppA is a multi-spanning membrane protein with nine transmembrane helices, supported by at least three TM prediction tools, matching the canonical architecture of signal peptide peptidase family members [42]. The predicted topology positions the N-terminus in the ER lumen and the C-terminus in the cytosol, consistent with experimentally validated eukaryotic SPP/SPPL topologies and reflecting the inverted membrane orientation relative to presenilins described in previous structural and functional studies [31,40].

Inspection of the predicted transmembrane regions identified all three hallmark motifs characteristic of SPP/SPPL-type intramembrane aspartyl proteases: the YD motif within TM6, the GxGD motif in TM7, and the QPALLY motif in the N-terminal portion of TM9. These motifs correspond to residues known to form the catalytic core of these enzymes [42], and mutation of either aspartate in the YD or GxGD motifs abolishes the proteolytic activity in multiple systems [43]. Moreover, the predicted luminal orientation of the loop between TM6 and TM7 is also consistent with the experimentally determined topology of eukaryotic SPPs [31]. Finally, the presence of the QPALLY motif in TM9 matches the conserved structural features observed in both SPP/SPPLs and presenilins, where its close spatial proximity to the catalytic aspartyl residue in TM6 contributes to shaping the active site environment [44,45].

Together, these observations strongly support that *P. rhodozyma* SppA is an intramembrane aspartyl protease within the SPP/SPPL family, consistent with a role in membrane protein processing.

### 2.2. SPPA Gene Mutation in P. rhodozyma

To evaluate whether the *P. rhodozyma SPPA* gene encodes an intramembrane protease required for Sre1 activation, as SppA does for the SREBP ortholog SrbA in *Aspergillus* species, Δ*sppA* mutants would be expected to display phenotypes similar to that from Δ*sre1* and Δ*stp1* mutants. These include hypersensitivity to clotrimazole and CoCl_2_, as well as reduced pigmentation in a *cyp61*^−^ or Sre1N context. Azoles such as clotrimazole inhibit Cyp51, a cytochrome P450 monooxygenase involved in sterol biosynthesis, thereby blocking ergosterol production [54], whereas CoCl_2_ is widely used as a hypoxia-mimicking compound [55].

**Figure 3:**
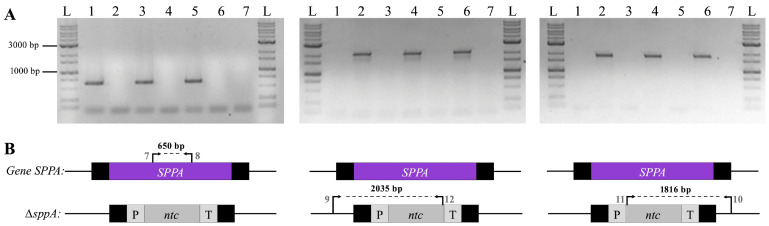
*SPPA* gene replacement in *P. rhodozyma* strains. (**A**) PCR analysis using genomic DNA from strains WTF (lane 1), Δ*sppA* (lane 2), *cyp61*^−^ (lane 3), *cyp61*^−^Δ*sppA* (lane 4), Sre1N (lane 5), Sre1NΔ*sppA* (lane 6), and a negative control without DNA (lane 7). DNA fragment sizes were determined by comparison with the GeneRuler 1 kb Plus DNA ladder (L). (**B**) Diagrams beneath each gel show the predicted PCR fragments and their expected sizes. Arrows indicate primer locations, numbered as in Table S2. Colors denote the *SPPA* gene (purple), the nourseothricin resistance cassette (gray), and the flanking homologous sequences of the *SPPA* locus for homologous recombination (black).

To test this possibility, Δ*sppA* mutants were constructed in the three strain backgrounds: CBS.*FLAG*.*SRE1*, CBS.*cyp61*^−^.*FLAG*.*SRE1*, and CBS.*FLAG*.*SRE1N* strains (hereafter referred to as WTF, *cyp61*^−^, and Sre1N, respectively), yielding strains CBS.*FLAG*.*SRE1*.∆*sppA*, CBS.*cyp61*^−^.FLAG.*SRE1*.∆*sppA*, and CBS.*FLAG*.*SRE1N*.∆*sppA* (hereafter referred to as Δ*sppA*, *cyp61*^−^Δ*sppA* and Sre1NΔ*sppA*, respectively). Each mutant was generated by transforming the corresponding parental strain with the linear deletion fragment released from plasmid pBS-Δ*sppA^ntc^*. This fragment contained the 5′ and 3′ flanking regions of the *SPPA* locus to promote homologous recombination, along with a nourseothricin resistance marker (Figure S3). The correct replacement of the *SPPA* gene was confirmed by PCR using primer pairs designed to verify the loss of the native gene and the correct integration of the resistance marker at both recombination junctions (Figure 3).

**Figure 4:**
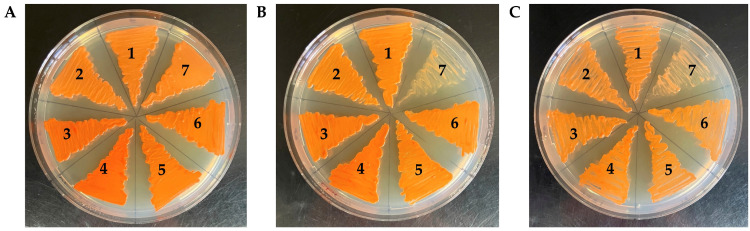
Phenotypic analysis of Δ*sppA* mutants and parental strains. Plates (1.5% agar) were incubated for 5 days at 22 °C. Cultures on (**A**) YM medium and YM medium supplemented with (**B**) clotrimazole (0.15 µg/mL) or (**C**) cobalt chloride (400 µM). Strain number: WTF (1), Δ*sppA* (2), Sre1N (3), Sre1NΔ*sppA* (4), *cyp61*^−^ (5), *cyp61*^−^Δ*sppA* (6), and a control strain Δ*sre1* (7).

To assess the phenotypes of the generated strains, their growth was evaluated on YM agar plates supplemented with or without clotrimazole or CoCl_2_, including strain Δ*sre1* as a control (Figure 4). All Δ*sppA* mutants grew in the presence of both compounds, in clear contrast to the Δ*sre1* strain, which, as previously described, failed to grow under these conditions. These results indicate that deletion of the *SPPA* gene does not impair the cellular response to ergosterol depletion or hypoxia-mimicking stress in *P. rhodozyma*. Moreover, no visible pigmentation differences were observed in the Δ*sppA* mutants derived from either the *cyp61*^−^ or Sre1N strains.

The observed phenotypic behavior of the Δ*sppA* mutants differs from what is expected when Sre1 activation is compromised. In fungi where SREBP orthologs have been well characterized, such as *S. pombe* [18] and *C. neoformans* [22,55], Sre1 activation is induced by azoles [54] or by CoCl_2_ [55], and Δ*sre1* mutants fail to grow in the presence of these compounds. A similar scenario has been described in *P. rhodozyma*, where Δ*sre1* and Δ*stp1* mutants exhibit hypersensitivity to azoles and CoCl_2_, being unable to grow when exposed to these treatments, unlike their parental strains [29,30]. These mutations also reduce sterol and carotenoid content in a *cyp61^−^* mutant strain that overproduces both metabolites [4]. Later work showed that Sre1 is constitutively activated in the *cyp61^−^* strain, suggesting that the *cyp61*^−^ mutation generates the physiological conditions that activate Sre1, and that deletion of *STP1* suppresses this activation, abolishing the carotenoid overproduction phenotype in this yeast [30]. Furthermore, the Sre1N strain, which expresses only the N-terminal transcription factor domain of Sre1, exhibits increased carotenoid production [29], and this Sre1 fragment complements the Δ*stp1* mutation [30,56]. Although this truncated version is interpreted as the active form of Sre1, it may still require additional processing or stabilization to achieve full functionality, raising the possibility that an SPP-type protease could contribute to such processing. However, all ∆*sppA* mutants generated in this work grew normally in the presence of clotrimazole and CoCl_2_, and no visible defects in pigmentation were detected in either the *cyp61*^−^ or Sre1N backgrounds. The absence of hypersensitivity to these compounds or pigment reduction (two hallmark phenotypes associated with impaired Sre1 activation) strongly suggests that SppA does not participate in Sre1-dependent responses in *P. rhodozyma*. Altogether, the phenotypic behavior of the Δ*sppA* mutants clearly contrasts with that of Δ*sre1* and Δ*stp1* strains, indicating that, unlike the situation described in *Aspergillus* species, an SPP-type intramembrane protease is not required for Sre1 activation in this basidiomycete. Although direct detection of Sre1 processing (for example by immunoblotting) would provide additional confirmation, in *P. rhodozyma* Sre1 activity is functionally reflected by growth in the presence of clotrimazole or CoCl_2_. The Δ*sppA* strains retained this Sre1-dependent phenotype and clearly differed from the Δ*sre1* mutant, indicating that Sre1-mediated responses occur normally in the absence of SppA.

Together, these results indicate that SppA is not required for Sre1 activation in *P. rhodozyma*, and the absence of additional SPP-like genes in the genome further supports the likelihood that an SPP-type protease does not participate in this regulatory process.

### 2.3. Transcriptional Effects of SPPA Gene Deletion in Distinct Genetic Backgrounds

**Figure 5:**
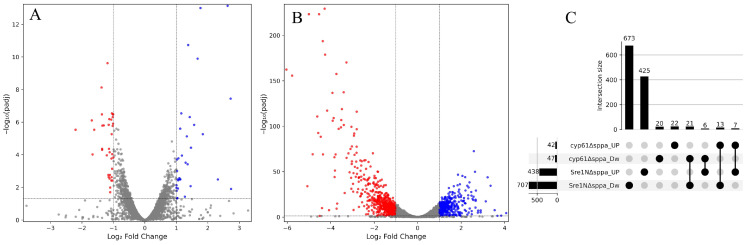
Differential gene expression in *cyp61*^−^Δ*sppA* and Sre1NΔ*sppA* mutants relative to their parental strains *cyp61*^−^ and Sre1N, respectively. Transcriptional changes resulting from *SPPA* deletion in the (**A**) *cyp61*^−^ and (**B**) Sre1N backgrounds. Dashed lines in volcano plots indicate the thresholds used to define differentially expressed genes (DEGs): |log_2_FC| ≥ 1.0 and adjusted *p*-value (padj) ≤ 0.05. (**C**) UpSet plot displaying background-specific and shared downregulated (Dw) and upregulated (Up) DEGs. Numbers above or at the left of bars indicate the number of DEGs in each category.

To support the phenotypic results suggesting that SppA is not involved in Sre1-dependent regulation in *P. rhodozyma*, transcriptomic analyses of the Δ*sppA* mutants were performed in *cyp61*^−^ [30] and Sre1N [29] genetic backgrounds in which Sre1 is in its active state. In *cyp61*^−^, Sre1 activation arises as a consequence of the physiological conditions associated with the *cyp61*^−^ mutation. In contrast, strain Sre1N expresses only the N-terminal transcription factor domain of Sre1, which would not require further proteolytic activation. A comparison of the transcriptomes of Δ*sppA* mutants and their corresponding parental strains revealed a marked difference in the number of differentially expressed genes (DEGs) between the two genetic backgrounds. Specifically, 89 DEGs were identified in the *cyp61*^−^ background, whereas 1145 DEGs were detected in the Sre1N background (Figure 5A,B). In addition, the proportion of downregulated DEGs differed between backgrounds, accounting for 52% in *cyp61*^−^ and 62% in Sre1N. Only a small number of DEGs were shared between the two backgrounds. Most of these shared DEGs were downregulated in both backgrounds (21 genes), whereas a second subset (13 genes) exhibited opposite expression patterns, being upregulated in *cyp61*^−^ and downregulated in Sre1N (Figure 5C).

Considering that the *cyp61*^−^ background provides a physiological context for Sre1 activation and given that Sre1 is known to positively regulate its own expression upon activation, we first examined Sre1 expression under this context [5]. Therefore, if SppA were involved in Sre1-dependent regulation in *P. rhodozyma*, the Δ*sppA* mutation would be expected to alter the expression levels of *SRE1* and of its direct target genes in the *cyp61*^−^ background. However, as shown in Table 1, the *SRE1* gene (ID g4728) did not exhibit significant changes in expression as a result of the Δ*sppA* mutation under the tested conditions, suggesting that the Δ*sppA* mutation does not prevent Sre1 activation. This interpretation is further supported by the absence of significant transcriptional changes in thirteen genes previously identified as direct Sre1 targets in this yeast through ChIP-exo analyses [5].

**Figure 6:**
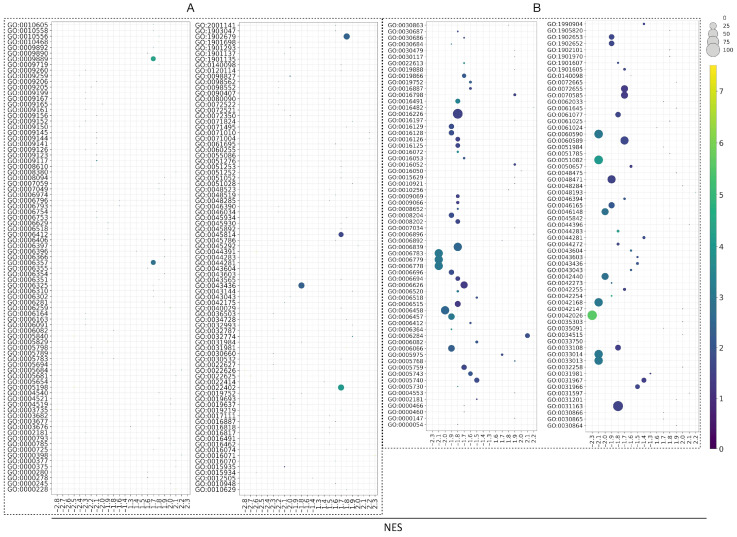
Gene Ontology enrichment analysis of transcriptional changes resulting from *SPPA* deletion. Gene Ontology enrichment analysis in the (**A**) *cyp61*^−^ and (**B**) Sre1N backgrounds. NES indicates normalized enrichment scores. Circle size represents the percentage of genes with significantly high fold changes within each GO term, and circle color corresponds to −log_10_ of the adjusted *p*-value (padj). GO terms were considered significantly enriched when padj was ≤0.05.

Next, we performed enrichment analyses in both genetic backgrounds (Figure 6). The number of significantly enriched GO terms was higher in the *cyp61*^−^ background than in Sre1N (166 and 127 terms, respectively). In *cyp61*^−^, normalized enrichment scores (NES) ranged from −2.8 to 2.3, whereas in Sre1N, they ranged from −2.3 to 2.2. A major difference between the two genetic backgrounds was observed in the percentages of genes with significantly high fold changes within enriched GO terms. In the *cyp61*^−^ background, this proportion was zero for most GO terms, with only six terms reaching values of at least 30%. Among these, three terms reached 50%: cell cycle process (GO:0022402), negative regulation of RNA biosynthetic process (GO:1902679), and oxoacid metabolic process (GO:0043436), while three additional terms reached 33.3% each: regulation of biosynthetic process (GO:0009889), regulation of transcription by RNA polymerase II (GO:0006357), and negative regulation of gene expression, epigenetic (GO:0045814). These processes are not typical SREBP targets, supporting that SppA is not related to Sre1 activation in *P. rhodozyma.* The remaining enriched GO terms with significantly high fold changes exhibited proportions of 1.8% or lower.

**Figure 7:**
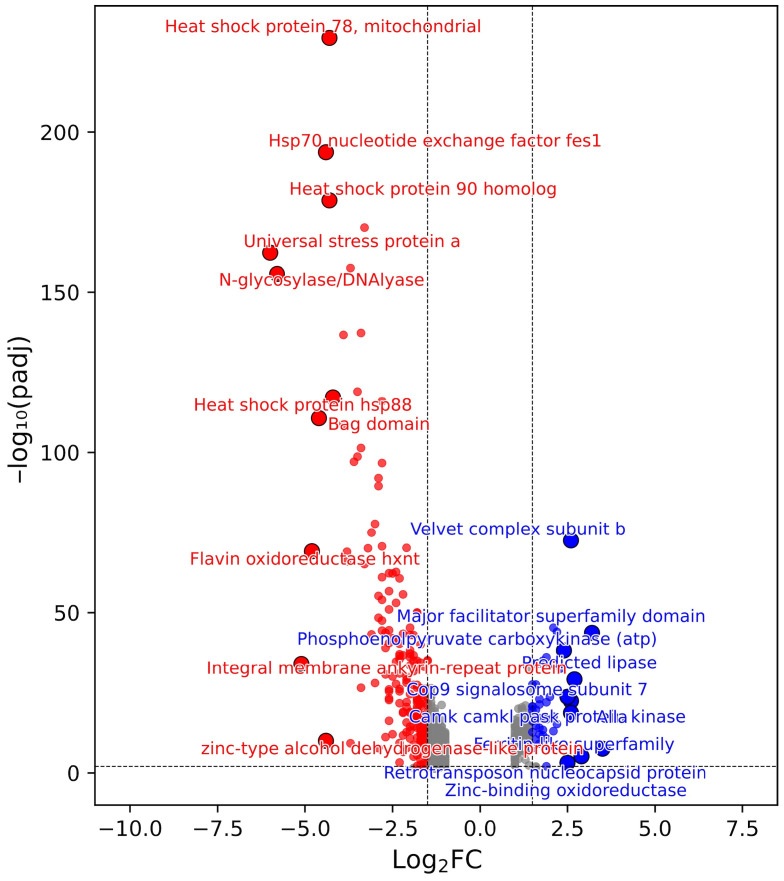
Transcriptional changes resulting from the *SPPA* gene deletion in the Sre1N background. Volcano plot showing differentially expressed genes (DEGs) defined using thresholds of |log_2_FC| ≥ 1.5 and adjusted *p*-value (padj) ≤ 0.01 represented by dashed lines. Only the top 10 downregulated (red) and upregulated (blue) genes are labeled.

In contrast, the Sre1N background displayed a larger number of GO terms with significantly high fold changes. Among these, 43 GO terms had proportions of at least 20%, including iron-sulfur cluster assembly (GO:0016226), metallo-sulfur cluster assembly (GO:0031163), protein refolding (GO:0042026), unfolded protein binding (GO:0051082), and ATPase regulator activity (GO:0060590), each reaching proportions of at least 75%. These patterns indicate that transcriptional changes in the *cyp61*^−^ background are predominantly moderate in magnitude, whereas in the Sre1N background, a larger proportion of genes exhibit high fold changes. Notably, the most pronounced changes in the Sre1N background (reflected by higher absolute NES values, lower adjusted *p* values, and a greater proportion of genes with significantly high fold changes), were observed for protein refolding (GO:0042026), unfolded protein binding (GO:0051082), and ATPase regulator activity (GO:0060590), all of which were markedly downregulated in the Δ*sppA* mutant. Consistent with these enrichments, sixteen chaperone genes were downregulated in the Sre1N background, four of which ranked among the ten most strongly downregulated genes: Hsp70 nucleotide exchange factor Fes1, heat shock protein 78 (mitochondrial), heat shock protein 90 homolog, and heat shock protein Hsp88. Additionally, a universal stress protein was among the ten most downregulated genes (Figure 7).

Together, these results suggest that SppA may be associated with a protein stress-related response in *P. rhodozyma* that becomes apparent specifically in the Sre1N background. In the Sre1N context, the likely deregulated activity of Sre1 may increase protein folding demand, thereby revealing a functional requirement for SppA under conditions of elevated proteotoxic stress. These observations are particularly intriguing in light of previous studies showing that signal peptide peptidases play a role in ER quality control by functionally linking ER-associated degradation (ERAD) and the unfolded protein response (UPR) [31,57]. ERAD serves as a central protein degradation pathway responsible for the recognition and proteasomal degradation of misfolded or unassembled proteins originating in the ER [58], whereas the UPR constitutes a signaling network triggered by ER protein folding stress, functioning to restore ER homeostasis by adjusting protein folding capacity [58]. In higher eukaryotes, the UPR operates through three major signaling modules: ATF6, PERK, and IRE1, which together remodel gene expression programs to recover ER proteostasis [59]. Among these branches, IRE1 represents the most evolutionarily conserved UPR sensor, described in yeast and in higher eukaryotes [60,61]. Within this framework, IRE1 is an ER-resident kinase/RNase that senses unfolded proteins and activates the transcription factor XBP1s (X-box binding protein 1) through an unconventional splicing of XBP1 pre-mRNA [59]. In contrast, the unspliced XBP1 mRNA encodes XBP1u, which negatively regulates UPR by promoting the proteasomal degradation of XBP1s [57]. In this context, SPP has been shown to form a multiprotein ERAD complex with Derlin1 and the E3 ubiquitin ligase TRC8, which mediates the intramembrane cleavage of XBP1u and targets it for proteasomal degradation [57]. Through this mechanism, SPP indirectly promotes UPR signaling by relieving the inhibitory effect of XBP1u on XBP1s. Consistent with this framework, the strong downregulation of protein refolding and unfolded protein binding processes, together with the repression of multiple chaperone-encoding genes observed in the Δ*sppA* mutant, is compatible with a role for SppA in protein-stress regulation under the Sre1N background.

**Figure 8:**
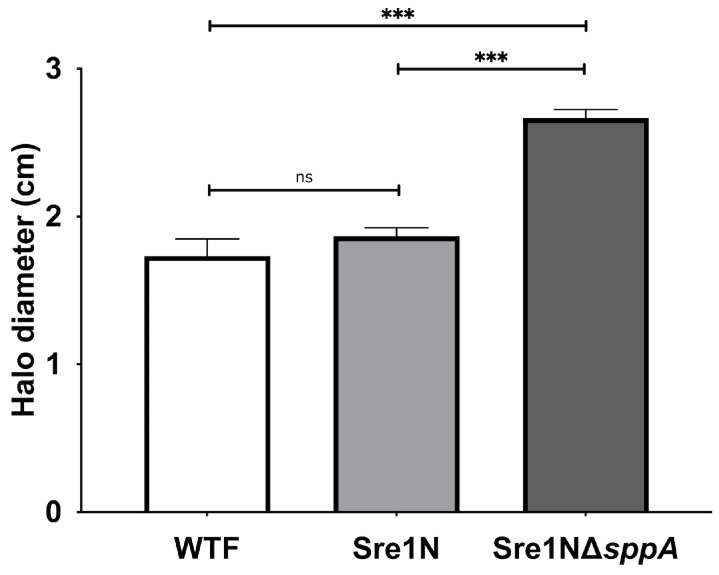
DTT sensitivity assay. Cell lawns of WTF, Sre1N and Sre1NΔ*sppA* strains were prepared on YM-1.5% agar plates. A sterile filter disk was placed at the center of each plate, and 10 µL of 1.0 M DTT was applied directly onto the disk. Plates were incubated at 22 °C for three days, and halo diameters were recorded. Data represent the mean of three independent replicates, and error bars indicate standard deviation. Statistical analysis was performed using one-way ANOVA followed by Tukey’s post hoc test. Adjusted *p* value: *** *p* < 0.001 (ns: not significant).

Finally, because the Δ*sppA* mutant displayed a transcriptomic signature consistent with altered protein-stress responses specifically in the Sre1N background, such as UPR as suggested by the marked downregulation of the GO terms protein refolding (GO:0042026) and unfolded protein binding (GO:0051082), together with several chaperone encoding genes ranking among the most strongly downregulated genes, we tested the sensitivity of strain Sre1NΔ*sppA* to dithiothreitol (DTT), a reducing agent that perturbs disulfide bond formation in the ER and compromises protein folding [62]. Under these conditions, this strain exhibited greater sensitivity than both the parental Sre1N and wild-type strains (Figure 8 and Figure S4), supporting a role for SppA in coping with ER-associated protein stress in the Sre1N background.

## 3. Materials and Methods

### 3.1. Identification and Bioinformatic Characterization of the P. rhodozyma SPPA Gene

In general, bioinformatic analyses were conducted with Geneious Prime^®^ 2025.1.3 and online available tools.

To identify the *P. rhodozyma* signal peptide peptidase encoding gene, protein sequences annotated as signal peptide peptidase were downloaded from the NCBI Identical Protein Groups database. The dataset was filtered by sequence length, retaining proteins between 200 and 800 amino acids, which represented approximately 96% of the downloaded sequences. Sequences whose descriptions included the terms “hypothetical”, “uncharacterized”, “partial”, “low quality protein”, “possible”, “predicted”, “probable”, or similar were excluded. Additionally, only sequences whose annotations included the terms “Spp”, “signal peptide peptidase”, “aspartic endopeptidase”, “presenilin”, or “minor histocompatibility antigen” were retained. This filtering resulted in a final dataset of 126,002 protein sequences (Table S1), which was used to build a local protein database in Geneious Prime^®^ 2025.1.3. The translated CDS previously predicted from the genome of the wild-type strain CBS 6938 [5,39] were compared against this local database using BLASTp [63] in Geneious Prime^®^ 2025.1.3, using default parameters and the option “Bin into ‘has hit’ vs. ‘no hit’”. Sequences classified as ‘has hit’ were subsequently subjected to a second BLASTp analysis against the same local database, retrieving a hit table and retaining the best hit per query. For this second analysis, hits were filtered using an e-value cutoff of ≤1 × 10^−10^ and a minimum query coverage of 70%. Under these criteria, a single candidate protein, g1388, was identified. In addition, a reciprocal BLASTp analysis was performed, comparing the filtered, downloaded dataset against the CBS 6938 predicted proteome, both with and without g1388. In this reciprocal approach, g1388 was the only sequence that consistently met the established threshold criteria, supporting its identification as the signal peptide peptidase candidate in *P. rhodozyma*. To validate its identity, the deduced protein sequence from the identified gene was subjected to BLASTp searches against the NCBI protein databases RefSeq and Swiss-Prot. To validate the exon-intron organization of the identified *locus*, RNA-seq reads from the wild-type strain (PRJNA966154) were mapped to a 10,000 bp genomic region containing the candidate CDS using the STAR 2.7.10a mapper [64] in Geneious Prime^®^ 2025.1.3 using default parameters. For phylogenetic analysis, protein sequences from the SPP/SPPL family from *S. cerevisiae*, *S. pombe*, *D. rerio*, *H. sapiens* and *M. marisnigri* were retrieved using the protein accession codes reported in [31]. In addition, sequences corresponding to the *A. nidulans* SppA homolog, homologous proteins from *M. musculus*, and the aspartic endopeptidase from *M. frigida*, which was identified as the best BLASTp hit against the RefSeq database for *P. rhodozyma* SppA, were included in the analysis. Protein sequences were aligned using MAFFT v7.490 [65] with the L-INS-i algorithm. Phylogenetic reconstruction was performed in Geneious Prime^®^ 2025.1.3 using the Jukes-Cantor genetic distance model and the Neighbor-Joining method with 1000 bootstrap replicates. The archaeal presenilin/SPP homolog (PSH) from *M. marisnigri* [66] was included as an outgroup.

Prediction of protein domains was performed using InterPro [67] with default parameters. Family classifications were obtained from PANTHER [50], Pfam [51], and SMART [52], and transmembrane segments were predicted using PHOBIUS [46], TOPCONS [47], MINNOU [48] and TMHMM [49]. The conserved YD, GxGD, and QPALLY motifs located in TM6, TM7, and TM9, respectively, in SppA were manually identified. The DeepLoc-2.1 web server [68] was used to predict subcellular localization.

### 3.2. Microorganisms and Culture Conditions

The strains and plasmids used in this work are listed in Table 2. *P. rhodozyma* strains were routinely grown at 22 °C in YM medium (0.3% yeast extract, 0.3% malt extract, and 0.5% peptone) supplemented with 1% glucose, using orbital shaking to maintain aeration. Yeast transformants were selected on YM plates (1.5% agar) containing 50 µg/mL nourseothricin. For phenotypic assays, strains were streaked onto YM agar plates supplemented with clotrimazole (0.15 µg/mL) or CoCl_2_ (400 µM), at concentrations that did not impair the growth of the parental CBS 6938 strain. Plates were incubated at 22 °C for 5 days before evaluation.

For dithiothreitol (DTT) stress assays, cells were spread as a lawn on YM-agar plates, and a sterile filter disk was deposited at the center of the plate. A 10 µL drop of 1.0 M DTT was placed on the disk, and the plates were incubated at 22 °C for three days.

*Escherichia coli* strains used for plasmid propagation were cultivated in lysogeny broth (LB) at 37 °C with shaking. LB-agar plates were supplemented with 100 µg/mL ampicillin for plasmid maintenance, and 32 µg/mL X-gal (5-bromo-4-chloro-3-indolyl-β-D-galactopyranoside) was added for recombinant clone selection [69]. Recombinant *E. coli* clones carrying plasmids generated in this work were identified by direct colony PCR.

### 3.3. Nucleic Acid Purification and PCR Analysis

Genomic DNA from *P. rhodozyma* was obtained by bead-assisted mechanical lysis. Cell pellets were suspended in 600 µL TE buffer (25 mM Tris-HCl, 10 mM EDTA, pH 8.0) and mixed with an equal volume of phenol: chloroform: isoamyl alcohol (25: 24: 1, *v*/*v*/*v*) and 0.5 mm glass beads (100 µL). Disruption was carried out in a Mini-beadbeater-16 (BioSpec Products Inc., Bartlesville, OK, USA) for three min. Following centrifugation (18,440× *g*, 5 min), the aqueous phase was recovered and re-extracted with chloroform: isoamyl alcohol (24: 1, *v*/*v*) to remove residual phenol. DNA was precipitated with cold absolute ethanol (1 mL), incubated at −20 °C for one hour, collected by centrifugation (18,440× *g*, 10 min), air-dried, and finally dissolved in 100 µL sterile water.

Plasmid DNA from *E. coli* was purified using the GeneJET Plasmid Miniprep Kit (Thermo Fisher Scientific Inc., Waltham, MA, USA) according to the supplier’s protocol.

PCR reactions contained 1X PCR buffer (500 mM KCl, 200 mM Tris-HCl, pH 8.4), 2 mM MgCl_2_, 0.2 µM each dNTP, 1 µM each primer, 1 U of *Pfu* DNA polymerase, and approximately 10 ng of template DNA. Amplifications were conducted in a 2720 thermal cycler (Applied Biosystems, Foster City, CA, USA), under the following program: 94 °C for 3 min; 35 cycles of 94 °C for 30 s, 55 °C for 30 s, and 72 °C for 2 min; followed by a final extension step at 72 °C for 10 min. Reactions were maintained at 4 °C until use. Primers sequences are listed in Table S2.

### 3.4. Plasmid Construction and Yeast Transformation

All plasmids used in this work are listed in Table 2. To delete the *SPPA* gene in *P. rhodozyma* strains, plasmid pBS-Δ*sppA^ntc^* was constructed. The regions flanking the *SPPA* coding sequence (642 bp upstream and 623 bp downstream) were amplified by PCR from genomic DNA of the wild-type strain CBS 6938 (Figure S3). These fragments were fused using overlap extension PCR, introducing an *Hpa*I restriction site between them. The resulting product was then cloned into the *Eco*RV site of pBluescript SK-. Then, a nourseothricin resistance cassette, previously amplified from pBS-*nat* [30], was inserted into the generated *Hpa*I site. The generated pBS-Δ*sppA^ntc^* plasmid was digested with *Spe*I and *Xba*I to release the linear donor fragment, which was used for the targeted replacement of the *SPPA* gene in strains WTF, *cyp61*^−^, and Sre1N. Gene deletion was achieved by double homologous recombination (Figure S3), yielding strains Δ*sppA*, *cyp61*^−^Δ*sppA*, and Sre1NΔ*sppA*, respectively (Table 2).

Transformation of *P. rhodozyma* was performed by electroporation as described previously [70,71]. Electrocompetent cells were prepared from cultures in exponential phase and electroporated using a Gene Pulser Xcell^TM^ (BioRad Laboratories Inc., Hercules, CA, USA) under the following settings: 125 mF, 600 Ω, and 0.45 kV. For each transformation, 10–15 µg of linear donor DNA obtained from the corresponding plasmid digestion was used. Transformants were selected on YM agar plates containing nourseothricin (50 µg/mL). Gene replacement was verified by PCR using primer sets designed to confirm correct cassette integration at both flanking regions.

### 3.5. RNA Extraction, Library Preparation, and Sequencing

Total RNA was extracted from three independent cultures of each strain harvested at the late exponential growth phase (30 h). Cell pellets were suspended in lysis buffer (2 mM sodium acetate, pH 5.5; 0.5% SDS; 1 mM EDTA in 0.1% DEPC-treated water) containing 0.5 mm glass beads and subjected to mechanical disruption for 3 min in a Mini-Beadbeater-16. TRI Reagent (800 µL; Thermo Fisher Scientific Inc., Waltham, MA, USA) was then added and homogenization was repeated for an additional 3 min. Phase separation was performed by chloroform addition, followed by incubation at room temperature for 10 min and centrifugation at 18,440× *g* for 10 min at 4 °C. The aqueous fraction was recovered and RNA precipitated with isopropanol. RNA quantity and purity were assessed spectrophotometrically.

Library preparation and sequencing were carried out by the TCL Group (Santiago, Chile). In brief, mRNA was enriched using VAHTS mRNA Capture Beads 2.0 (Vazyme, Nanjing, China). Strand-specific libraries were generated with the VAHTS Universal V8 RNA-seq Library Prep Kit for MGI (Vazyme, Nanjing, China) and circularized using the MGIEasy Circularization Kit V2.0 (MGI, Shenzhen, China). Sequencing was performed on a DNBSEQ-G400 platform with the MGI sequencing kit DNBSEQ-G400RS High-throughput Sequencing Set (MGI, Shenzhen, China), producing 2 × 150 bp paired-end reads. Across samples, raw read counts ranged from 54 to 59 million (Table S3). Raw paired-end RNA-Seq reads were deposited in the NCBI SRA database under accession number PRJNA966154.

### 3.6. RNA-Seq Data Processing and Differential Expression Analysis

Raw paired-end RNA-Seq reads were processed with fastp v0.23.2 [72] to remove adapters and low-quality bases. Read-length thresholds were adjusted according to the sequencer output (≥50 bp for 150 bp reads, ≥40 bp for 100 bp reads, and ≥30 bp for 75 bp reads). Bases with Phred scores below 20 were trimmed and reads not meeting these criteria were discarded. fastp was also used to eliminate poly-G and poly-X artifacts and to maintain proper pairing between mates. Summary statistics for each library are provided in (Table S3).

Sequencing reads were mapped against the *P. rhodozyma* CBS 6938 reference genome (GenBank accession: GCA_014706385.1) using previously annotated gene models [5,39]. Alignment was carried out with Rsubread v2.18.3 [73] under default settings (seed-and-vote mapping, Phred+33 quality encoding, and reporting a single primary alignment per read pair). Resulting BAM files were processed with the Rsamtools package 2.24.1 [74] to sort, index and retain only properly paired high-quality alignments. Gene-level read counts were obtained with featureCounts using the corresponding GFF annotation. For each library, expression values were calculated as RPKM and TPM. Differential gene expression analysis was assessed in R with DESeq2 v1.40.1 [75]. Raw counts produced by featureCounts were imported and samples grouped according to the first character of each sample name. For each comparison, a DESeqDataSet was created using the design formula ~ Condition, defining a reference level to ensure consistent contrast direction. Standard DESeq2 procedures were used to estimate size factors, dispersion, and negative binomial model parameters. Wald tests were then applied to detect differential expression. Genes meeting the thresholds |log_2_FC| ≥ 1 and adjusted *p*-value (padj) ≤ 0.05 were considered significantly up- or down-regulated. Complete results for pairwise comparisons are included in Table S4.

Gene Ontology (GO) enrichment analysis was performed using a true Gene Set Enrichment Analysis (GSEA) framework based on ranked gene-level statistics [76]. Differential expression results were imported into R (version 4.5.1), retaining genes with valid identifiers and log_2_ fold-change (log_2_FC) values. Genes were ranked in decreasing order of log_2_FC to generate the ordered input required for GSEA. Custom GO annotations were generated directly from the dataset by mapping GO terms to gene identifiers (TERM2GENE), enabling enrichment analysis independent of organism-specific annotation packages. GO term names and definitions were retrieved using the GO.db and AnnotationDbi packages [77]. GSEA was conducted using the *GSEA* function in the clusterProfiler package [78], with gene set sizes restricted to 10–500 genes. Enrichment significance was evaluated by permutation testing, and multiple testing correction was performed using the Benjamini–Hochberg false discovery rate (FDR) method [79]. Enrichment results were summarized using normalized enrichment scores (NES), nominal *p*-values, and adjusted *p*-values. To further characterize enrichment drivers, leading-edge subsets were analyzed for each significantly enriched GO term, as defined in the original GSEA framework [76]. Genes with an absolute log_2_ fold change (|log_2_FC|) ≥ 2 were classified as highly differentially expressed, and both their number within each leading-edge subset and their percentage relative to the leading-edge size were calculated.

Plots were generated in Python 3 using pandas (version 2.2.3) [80], Matplotlib (version 3.9.2) [81], seaborn (version 0.13.2) [82], and NumPy (version 2.1.3) [83] libraries.

## 4. Conclusions

In this work, we identified and characterized a signal peptide peptidase homolog in *P. rhodozyma*, here named SppA, whose predicted sequence features, domain architecture, and phylogenetic placement are consistent with those described for signal peptide peptidases. Results from genetic, phenotypic, and transcriptomic analyses in backgrounds with constitutive Sre1 activity support that SppA is not required for activation of the SREBP homolog Sre1 and therefore does not function as a core component of the SREBP pathway in this yeast. However, loss of SppA in the Sre1N background was associated with transcriptional changes affecting genes involved in protein folding and unfolded protein binding, suggesting that SppA may participate in protein stress-related processes. Future studies will be required to elucidate the molecular mechanisms underlying this role and to determine how SppA contributes to protein homeostasis in *P. rhodozyma*.

## Figures and Tables

**Table 1:** Differential expression of Sre1 and its regulated genes in the *cyp61*^−^ background.

Gene	BLASTp Hit	log_2_	padj
g4728	Sterol regulatory element binding protein homolog (Sre1)	−0.5	1.2 × 10^−1^
g904	Sterol 24-C-methyltransferase	−0.8	3.6 × 10^−2^
g1347	Delta(14)-sterol reductase Erg24	−0.6	1.7 × 10^−1^
g2630	C-4 methylsterol oxidase Erg25	−1.0	1.1 × 10^−4^
g602	Methylsterol monooxygenase Erg25b	−0.3	5.5 × 10^−1^
g3516	Hydroxymethylglutaryl-coa synthase	−0.9	3.6 × 10^−1^
g3611	Lanosterol synthase	−0.5	7.1 × 10^−2^
g190	Lanosterol 14-alpha demethylase	−0.3	5.0 × 10^−1^
g580	Hypothetical protein_g580	−0.7	2.8 × 10^−2^
g5794	Delta(7)-sterol 5(6)-desaturase Erg3b	−0.5	8.0 × 10^−2^
g1512	hypothetical protein_g1512	0.3	4.9 × 10^−1^
g1377	3-hydroxy-3-methylglutaryl-coenzyme a reductase	−0.4	4.4 × 10^−1^
g4727	Orotate phosphoribosyltransferase	−0.2	7.1 × 10^−1^
g2015	Retrovirus-related pol polyprotein from transposon TNT 1-94	−0.1	1.0 × 10^0^

(comparison of strain *cyp61*^−^Δ*sppA* versus strain *cyp61*^−^).

**Table 2:** Strains and plasmids used in this work.

Strains/Plasmids	Description	Reference or Source
Strains		
*E. coli*		
DH5α	Amp^S^. Used for molecular cloning and plasmid maintenance.	[69]
*P. rhodozyma*		
CBS 6938	*P. rhodozyma* wild-type strain from which all mutants derive (Zeo^S^, Hyg^S^ and Ntc^S^)	ATCC 96594
CBS.*FLAG*.*SRE1*	Strain WTF. Mutant Zeo^S^, Hyg^R^ and Ntc^S^. The *SRE1* gene was replaced by a gene variant that expresses the Sre1 protein fused to the 3xFLAG epitope at its N-terminus, followed by the hygromycin B resistance cassette.	[30]
CBS.*FLAG.SRE1.*∆*sppA*	Strain Δ*sppA*. Mutant Zeo^S^, Hyg^R^ and Ntc^R^. The *SRE1* gene was replaced by a gene variant that expresses the Sre1 protein fused to the 3xFLAG epitope at its N-terminus, followed by the hygromycin B resistance cassette. The *SPPA locus* was replaced by the nourseothricin resistance cassette.	This work
CBS*.cyp61^−^.FLAG.SRE1*	Strain *cyp61^−^*. Mutant Zeo^R^, Hyg^R^ and Ntc^S^. The *SRE1* gene was replaced by a gene variant that expresses the Sre1 protein fused to the 3xFLAG epitope at its N-terminus, followed by the hygromycin B resistance cassette. The *CYP61 locus* was interrupted by the zeocin resistance cassette.	[30]
CBS.*cyp61^−^.FLAG.SRE1.*∆*sppA*	Strain *cyp61^−^*Δ*sppA*. Mutant Zeo^R^, Hyg^R^ and Ntc^R^. The *CYP61 locus* was interrupted by the hygromycin B resistance cassette. The *SRE1* gene was replaced by a gene variant that expresses the Sre1 protein fused to the 3xFLAG epitope at its N-terminus, followed by the hygromycin B resistance cassette. The *SPPA locus* was replaced by the nourseothricin resistance cassette.	This work
CBS.*FLAG.SRE1N*	Strain Sre1N. Mutant Zeo^R^, Hyg^S^ and Ntc^S^. The *SRE1* gene was replaced by a gene version that expresses Sre1N fused to the 3xFLAG epitope at its N-terminal, followed by the zeocin resistance cassette.	[29]
CBS.*FLAG.SRE1N.*Δ*sppA*	Strain Sre1NΔ*sppA*. Mutant Zeo^R^, Hyg^S^ and Ntc^R^. The *SRE1* gene was replaced by a gene version that expresses Sre1N fused to the 3xFLAG epitope at its N-terminal, followed by the zeocin resistance cassette. The *SPPA locus* was replaced by the nourseothricin resistance cassette.	This work
CBS.*sre1^−^*	Strain Δ*sre1*. Mutant Zeo^R^, Hyg^S^ and Ntc^S^. Approximately 90% of the coding region of gene *SRE1* was replaced by the zeocin resistance cassette.	[29]
Plasmids		
pBluescript SK- (pBS)	Cloning vector (ColE1 ori, Amp^R^, blue-white colony selection).	Agilent Technologies Inc., Santa Clara, CA, USA
pBS-*nat*	pBS containing the nourseothricin resistance cassette at the *Eco*RV site used for *P. rhodozyma* transformant selection.	[30]
pBS-Δ*sppA^ntc^*	pBS carrying the DNA fragment used for *P. rhodozyma* transformation. The construct contains 642 bp upstream and 623 bp downstream of the *SPPA* gene, flanking the nourseothricin resistance cassette at the *Eco*RV site of the vector. This fragment was used to delete the *P. rhodozyma SPPA* gene through homologous recombination.	This work

Zeo^S^/Zeo^R^: sensitive/resistant to zeocin. Hyg^S^/Hyg^R^: sensitive/resistant to hygromycin B. Nat^S^/Nat^R^: sensitive/resistant to nourseothricin. ColE1 ori: replication origin of *E. coli* ColE1 plasmid, Amp^S^/Amp^R^: sensitive/resistant to ampicilin. ATCC American Type Culture Collection.

## Data Availability

The datasets generated and analyzed during this study are available at the National Center for Biotechnology Information SRA database (Accession number PRJNA966154) and in the Supplementary Materials.

## Supplementary Materials

The following supporting information can be downloaded at: https://www.mdpi.com/article/10.3390/ijms27062628/s1.

## Abbreviations

The following abbreviations are used in this manuscript:

ATF6	Activating Transcription Factor 6
bHLH-ZIP	basic helix-loop-helix leucine zipper
CDS	Coding sequence
DEGs	Differentially Expressed Genes
Dsc	Defective for SREBP cleavage
DTT	dithiothreitol
ER	Endoplasmic Reticulum
ERAD	Endoplasmic Reticulum-Associated Degradation
GO	Gene Ontology
INSIG	Insulin induced gene
IRE1	Inositol-Requiring Enzyme 1
MAFFT	Multiple Alignment using Fast Fourier Transform
MVA	Mevalonate
NES	Normalized Enrichment Score
ORF	Open Reading Frame
padj	Adjusted *p*-value
PERK	PKR-like ER kinase
RNA-seq	RNA sequencing
RPKM	Reads Per Kilobase per Million mapped reads
S1P	Site-1 Protease
S2P	Site-2 Protease
SCAP	SREBP Cleavage-Activating Protein
SPP	Signal peptide peptidase
SPPL	Signal peptide peptidase like
SRE	Sterol Regulatory Element
SREBP	Sterol Regulatory Element-Binding Protein
TM	Transmembrane
TMHMM	TransMembrane Hidden Markov Model
TPM	Transcripts Per Million
UPR	Unfolded Protein Response
XBP1	X-box Binding Protein 1
XBP1s	Spliced XBP1
XBP1u	Unspliced XBP1
