# Supplementary figures and images for: Functional Characterization of a Signal Peptide Peptidase in Phaffia rhodozyma Reveals a Potential Role in Protein Stress Response but Not in Activation of the SREBP Ortholog Sre1
Source: Int J Mol Sci. 2026 Mar 13;27(6):2628. doi: 10.3390/ijms27062628 (PMC13026331; doi:10.3390/ijms27062628)

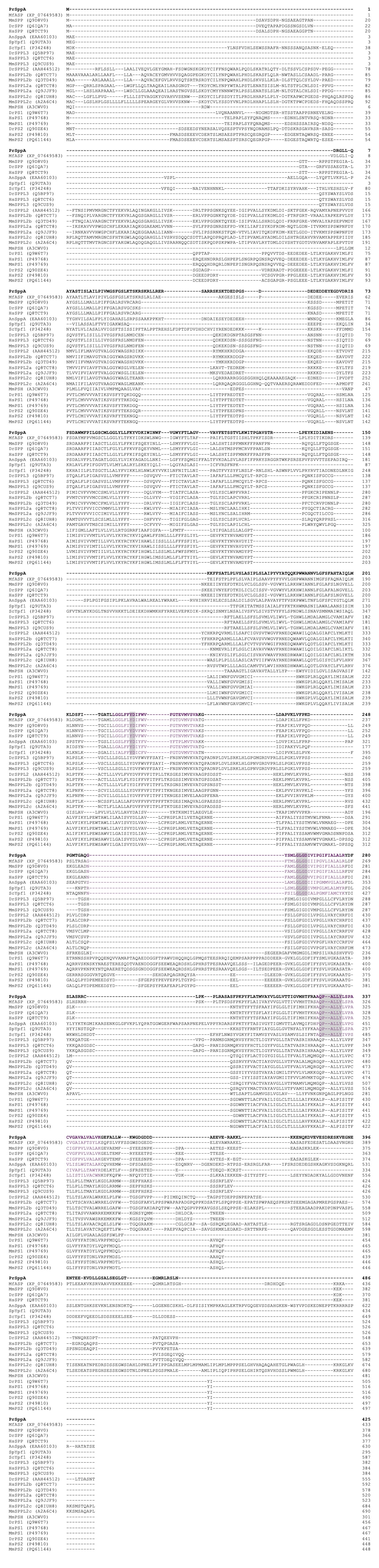

Supplement: Supplementary file 1 [file ijms-27-02628-s001.zip › Figure S1.jpg]

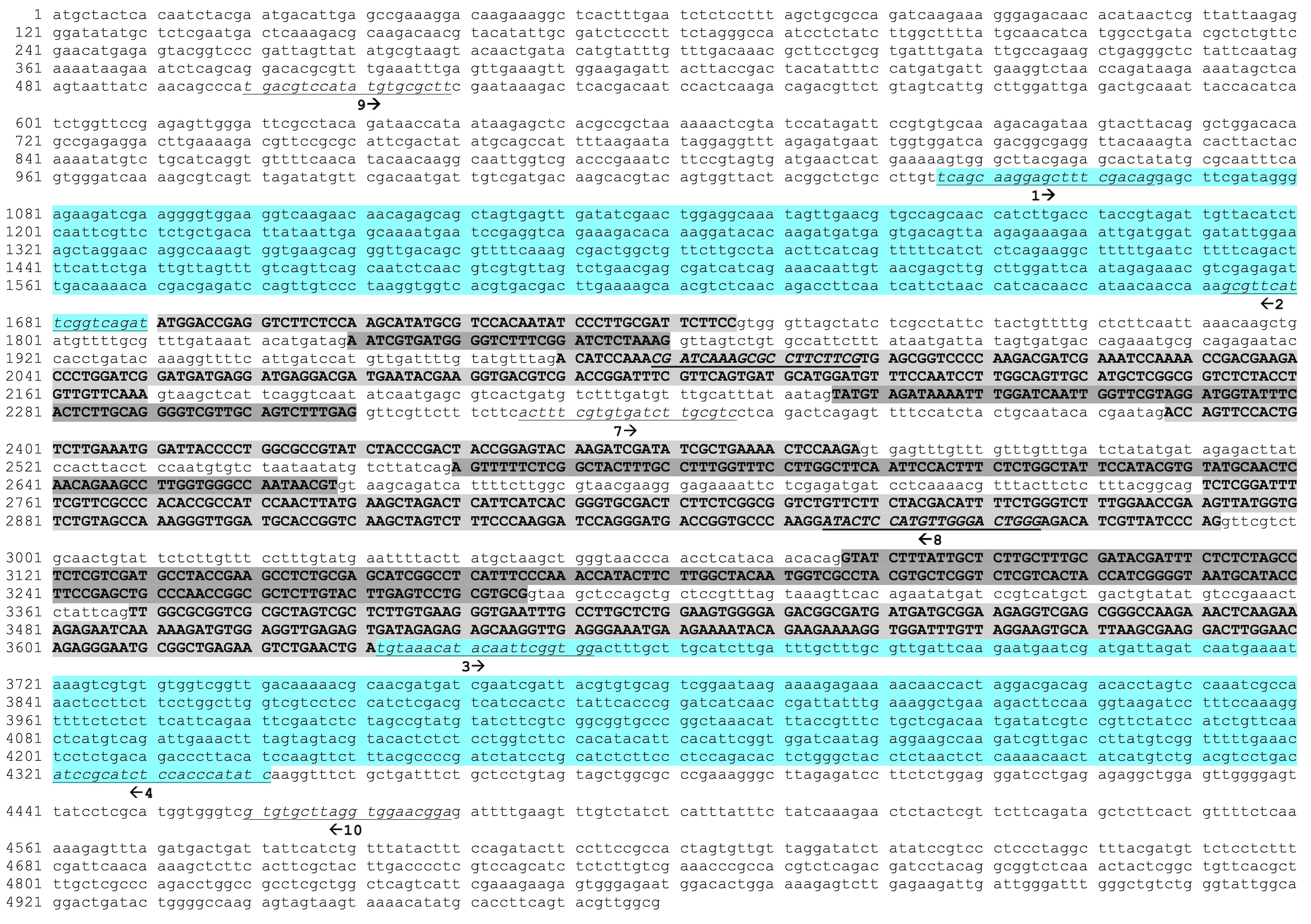

Supplement: Supplementary file 1 [file ijms-27-02628-s001.zip › Figure S2.jpg]

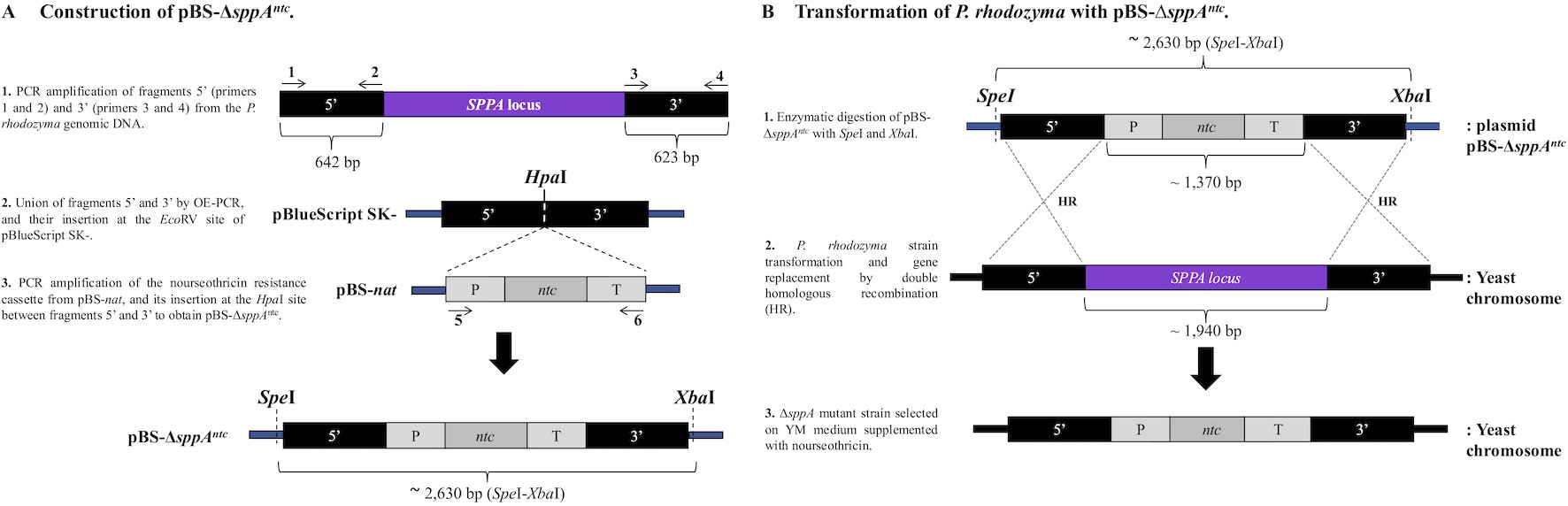

Supplement: Supplementary file 1 [file ijms-27-02628-s001.zip › Figure S3.jpg]

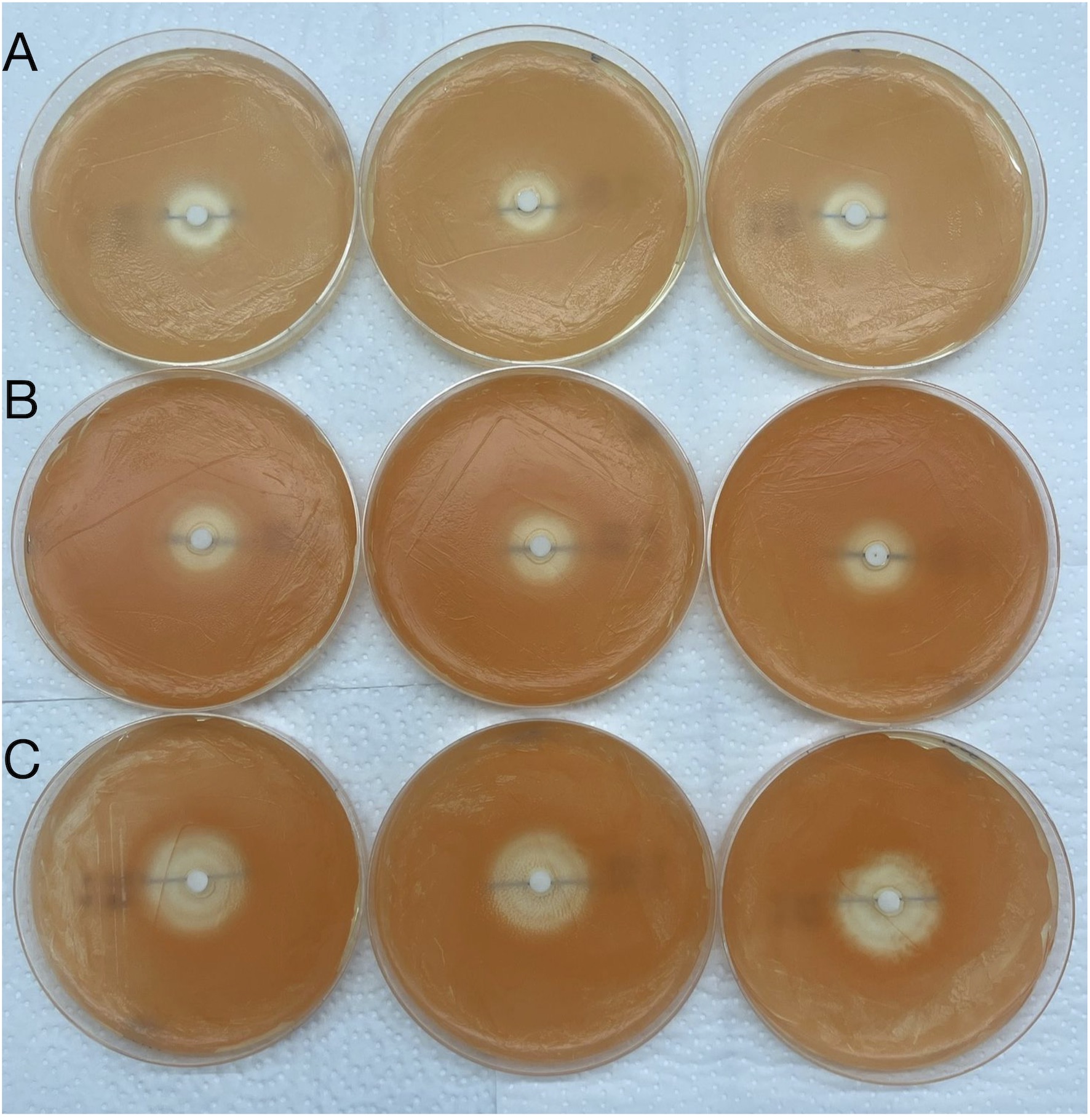

Supplement: Supplementary file 1 [file ijms-27-02628-s001.zip › Figure S4.jpg]
